# Egocentric networks and physical activity outcomes in Latinas

**DOI:** 10.1371/journal.pone.0199139

**Published:** 2018-06-18

**Authors:** Becky Marquez, Greg Norman, James Fowler, Kim Gans, Bess Marcus

**Affiliations:** 1 Department of Family Medicine & Public Health, University of California San Diego, La Jolla, California, United States of America; 2 School of Medicine, University of California San Diego, La Jolla, California, United States of America; 3 Department of Human Development and Family Studies, Storrs, Connecticut, United States of America; Columbia University, UNITED STATES

## Abstract

**Objective:**

Despite data linking the social environment to physical inactivity in Latinas, research on social network predictors of physical activity (PA) is limited. This study examined social network predictors of PA change in Latinas.

**Methods:**

Egocentric network data were collected from 102 adult Latinas (egos) participating in a randomized controlled PA intervention trial for underactive women. Moderate-to-vigorous PA (MVPA) was measured in minutes per week using the 7-Day PA Recall Interview and accelerometers at baseline and 12 months. Analyses characterized social network structure, composition, tie strength, homogeneity, and support for PA and determined the relationship between network characteristics and PA outcomes.

**Results:**

Networks had an average of four social ties (alters). Networks were high in density and transitivity and low in components, indicating high cohesion. Networks were primarily composed of females, Latinos, Spanish-speakers, and family members. Relationship ties were strong as evidenced by close living proximity, in-person contact, high emotional closeness, and long relationship duration. There was high homogeneity in demographics and PA behaviors. Multivariate analyses revealed that network size, familial ties, contact frequency, and ego-alter dissimilarities in age and running but similarities in walking, were associated with increased MVPA. Networks high in support for PA in the form of complimenting ego on exercise, taking over chores to allow ego to exercise, and co-participating with ego in exercise were also associated with greater MVPA.

**Conclusion:**

These findings contribute to better understanding interpersonal processes that may influence behavior change in a group with especially low levels of PA.

## Introduction

Latinas have disproportionately low levels of physical activity compared to non-Hispanic White women [1]. Effective interventions to increase physical activity are necessary to reduce the elevated risk and burden of chronic diseases associated with physical inactivity [2]. Physical activity interventions for Latinas have predominately focused on individual level change and have produced small to modest improvements [3]. Evidence suggests intervention effects are impacted by participants’ social networks [4]. To better account for interpersonal influences on physical activity interventions, it is important to first understand the social context of behavior change. Research on Latino social networks indicate that women have smaller and less diverse networks and receive less instrumental support than men [5]. These network characteristics have been linked to lower physical activity in non-Latinos [6–8]. Examination of social networks of Latinas participating in a physical activity intervention may provide insight into potential social influence on changes in physical activity.

Several studies have used social network analysis to understand how an individual’s physical activity is affected by people he/she knows and interacts with (social ties) [9–14]. The social network theoretical perspective asserts that characteristics of an individual’s network can be used to predict the attitudes and behaviors of that individual. Social network analysis provides a set of techniques used to quantify social relationships [15]. Research on social networks and physical activity has studied network structure, composition, tie strength, homogeneity, and support for physical activity and serves as the basis for the present study.

### Network structure

More cohesive social networks are smaller in size, have greater density and transitivity, and have fewer sub-group components [16]. Network size is the sum number of people in a social network. Having a larger social network is associated with more daily physical activity [14] and low social connectivity is linked to greater risk for physical inactivity [7]. Network density is defined as the proportion of people that know each other. Behaviors can spread more rapidly in dense networks [17]; however, they may be less open to new ideas from the outside. Hence, higher density could reflect stronger group norms [15]. One study found that adolescents with high density friendship networks were more likely to be sedentary than those with low density networks, and suggested involvement of exposure to normative attitudes and behaviors related to sedentary behavior [18]. Network transitivity is determined by the proportion of connected triads. It indicates that two individuals who are connected to one another are also connected to a third individual (e.g., a friend of a friend is your friend). Network members are more likely to be influenced by others that are connected to each other due to reinforcement of attitudes and behaviors [17]. Network components are subgroups that are disconnected from other members within the network. This could allow access to different points of view or novel sources of information. Components correspond to compartmentalized relationships which can serve distinct functions. For example, an individual may have three network components; each composed of family, friends, or co-workers that do not interact.

### Network composition

Network compositional characteristics of interest include age, gender, ethnicity, education, employment, and relationship types because they describe the network and may serve as predictors of behavioral influence [15]. Of these compositional characteristics, most research on social networks and physical activity in adults have focused on relationship types (e.g., family, friend, or neighbor ties). Several studies have linked friendship ties to greater physical activity across different racial/ethnic groups [19–22]. Longitudinal measurement of physical activity in women revealed that consistently active women were more likely than less active women to have a friend who was also active [23]. Friendship ties are a stronger predictor of physical activity levels than familial ties [19–21]. Some research suggests that familial ties hinder physical activity [24, 25]. Family networks may represent less resourceful networks [24] or more social roles strain [26, 27] at least in women.

### Network tie strength

Relationship strength also has implications for behavior change. Stronger relational ties indicate greater likelihood of being influenced by these ties [15]. Indicators of tie strength include duration of relationship, proximity of residence, frequency of social contact, and degree of emotional closeness [16]. Stronger ties may provide assistance when adopting a complex behavior. Having a greater number of social ties living nearby has been associated with more minutes per day of physical activity [14]. One study reported that individuals with higher contact (i.e., seeing or calling) with friends were more likely to meet the physical activity recommendation of 150 minutes per week of moderate-to-vigorous intensity [19]; suggesting social interactions allow for social exchanges that shape behavior. Another study found that the stronger the relationship between individuals and their social ties, the greater probability of being similar in physical activity engagement [13].

### Network homogeneity

Network homogeneity is another relevant concept in social network research. Social ties often resemble each other on characteristics such as demographics, attitudes, and behaviors [28]. Three reasons have been proposed to explain why people tend to cluster together based on shared characteristics. The first is homophily, which is the tendency of individuals to seek and associate with others like themselves [28]. Most of the evidence for homophily on physical activity comes from studies on adolescents, which find friends are selected based on similar participation in physical activity [11, 29, 30]. The second reason is shared environment, where individuals exposed to similar environments exhibit similar behaviors [31]. For example, individuals who live in neighborhoods where the built environment is not conducive to physical activity (e.g., limited parks and recreational facilities) may have comparable low levels of physical activity. The third reason is influence, whereby individuals become more similar overtime due to imitation, modeling, or shared norms. Longitudinal school-based studies have identified peer influence on physical activity where adolescents adopted physical activity behaviors of their friends [11, 30]. Hence, because connected individuals tend to share similarities, an individual’s attitudes and behaviors can be a reflection of their network.

### Network support

Social support is an important function of the network. Social support is the resources accessed from one’s social network. Types of social support include informational, emotional, and instrumental. Informational support is receiving advice or knowledge. Emotional support refers to communication of caring, understanding, and/or esteem. Instrumental support is assistance with needs through practical services or material aid. Those who receive social support particularly from family and friends are more likely to engage in regular physical activity [25, 32, 33]. Data on specific supportive behaviors related to physical activity in women are limited. Qualitative studies however indicate barriers to physical activity include family responsibilities as well as lack of encouragement and companionship for physical activity [27, 34, 35].

### Present study

**Fig 1:**
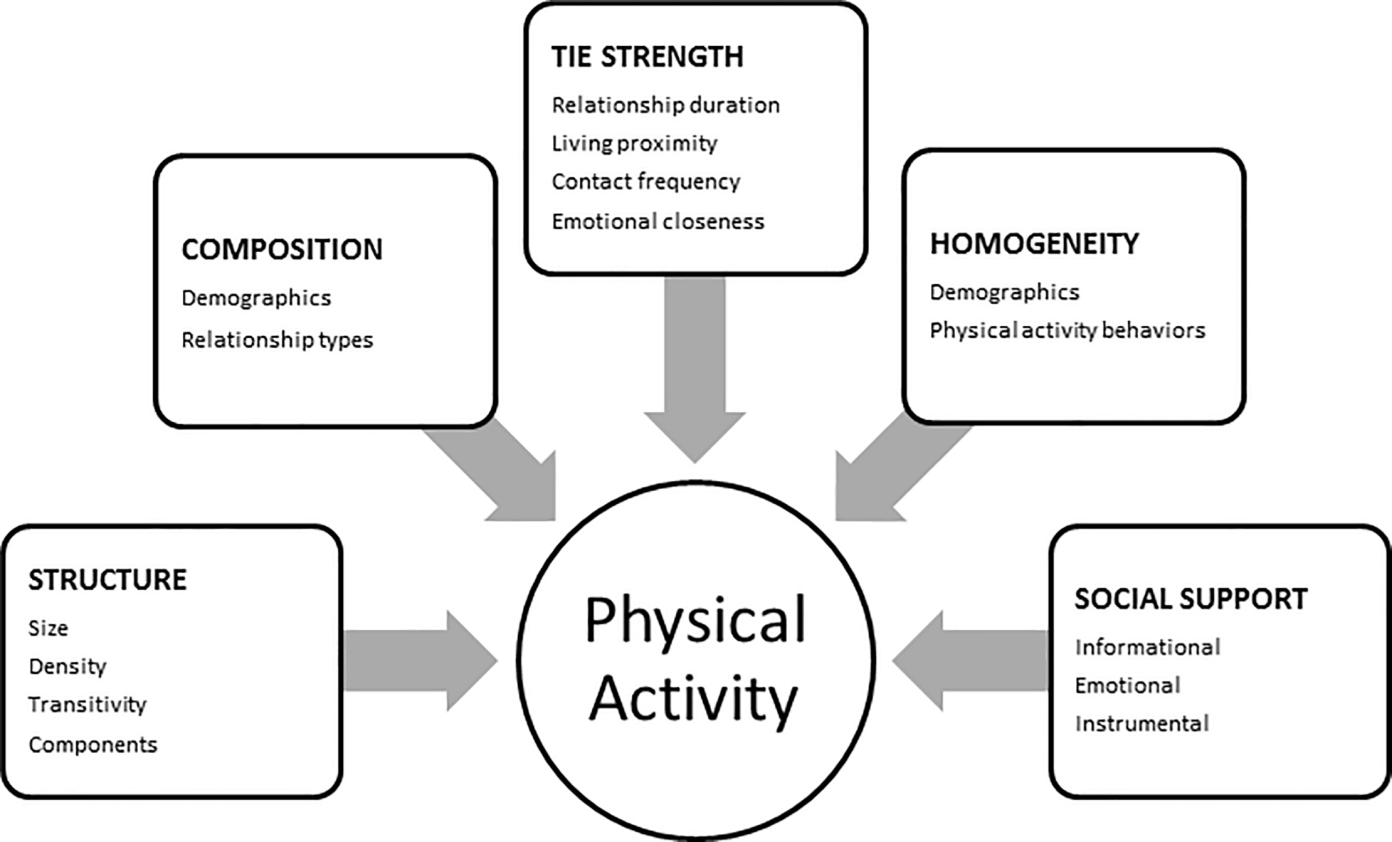
Model of social network predictors of physical activity.

The purpose of this study was to apply social network analysis to identify network level predictors of physical activity change in Latinas participating in a 12 month lifestyle intervention (Fig 1). An egocentric network approach was used where data were collected on specific social ties and their characteristics based on the respondent’s perspective. The approach is important because perception is a strong predictor of influence [36, 37]. The objectives of the study were to characterize social network structure, composition, tie strength, homogeneity, and support for physical activity and determine the relationship between these network characteristics and physical activity outcomes. We hypothesized that social networks that are less cohesive, more friendship-based, higher in contact, similar in physical activity, or supportive of physical activity would be associated with increased participant moderate-to-vigorous physical activity.

## Materials and methods

### Design

Participants were adult Latinas enrolled in the *Pasos Hacia la Salud* study [38, 39]; a 12 month randomized controlled trial (NCT 01834287). The study compared an internet-based physical activity intervention group to a wellness contact control group. Based on the Transtheoretical Model [40] and Social Cognitive Theory [41], the intervention emphasized behavioral change strategies including goal setting, self-monitoring, and problem solving. The main outcome of the study was change in self-reported minutes per week of moderate-to-vigorous physical activity (MVPA).

### Participants

To be eligible for the study, participants were required to be female, self-identify as Hispanic/Latino, be 18 to 65 years old, have a body mass index (BMI) of 18–45 kg/m^2^, have internet access, and not engage in more than 60 minutes per week of at least moderate intensity physical activity. Individuals were excluded from participation if they were pregnant, were unable to be physically active, or had a serious health or psychological condition. The study was approved by the University of California San Diego Institutional Review Board, and all participants gave written informed consent.

### Measures

#### Participant baseline characteristics

Demographic data were collected via a self-administered questionnaire. Participants provided information on age, income, education, employment status, nativity status, English language use, marital or cohabitation status, and children at home.

#### Egocentric network questionnaire

Egocentric network data were collected from participants at the 12 month visit via a face-to-face interview conducted in Spanish or English. Participants (egos) provided names of people (alters) with whom they discussed important matters in the past year [42]. Egos were asked to provide the name of alters in the order of extent to which they felt the most to least close. There was no limit on the number of alters that egos could name. Egos were asked to provide information about each alter. Information included alter age, gender, ethnicity, language of communication, and relationship type. Egos also reported on the relationship (ties) between alters [16].

Additional attribute data were collected for the primary three alters listed. Information included alter education, employment, and ego-alter tie strength. Egos reported the frequency of informational, emotional, and instrumental support for physical activity received from alters. The frequency of physical activity behaviors performed by each alter was also assessed. The egocentric network questionnaire was used to determine network structure, composition, tie strength, homogeneity, and support for physical activity.

#### Network structure and composition

Network size is the sum number of alters listed [15]. Network density is the proportion of alters with ties to each other divided by the total number of possible ties (range 0 to 1) [16]. Network transitivity is the number of connected triads divided by the total number of possible triads (range 0 to 1) [16]. Network components are the number of alter groups that were separated or disconnected from each other [16]. Network composition is determined by the proportion of each alter attribute reported [16].

#### Ego-alter tie strength

Four questions were used to assess tie strength. The duration of the ego-alter relationship was determined by the number of years the ego knew the alter. Living proximity was determined by whether the alter lived in the same household, same neighborhood, same city, different city, different county, different state, or different country. Contact frequency was determined by how often the ego had contact with the alter in-person. The response options were daily, weekly, monthly, less than monthly, and never. Emotional closeness was determined by whether the ego felt her relationship with the alter was extremely, very, moderately, somewhat, or not at all close.

#### Ego and alter physical activity behaviors

Physical activity behaviors, which could be performed with at least moderate intensity, were assessed for the ego-perceived alter and ego. The ego reported how often the alter engaged in walking for leisure, running or jogging, riding a bicycle or stationary bike, swimming, dancing, aerobic exercise (e.g., Zumba, elliptical), and playing team sports in the past year. The ego also reported how often she engaged in each type of physical activity. Response options were on a 5-point Likert scale: never, rarely, some of the time, most of the time, and always. For analysis, responses were combined to provide a dichotomous variable representing never plus rarely and some of the time, most of the time, plus always.

#### Network support for physical activity

Three types of support for physical activity received in the past year were measured. Each support item was selected based on the research team’s prior qualitative research with Latinas. Informational support was assessed by how often the alter gave the ego advice on exercise. Emotional support was assessed by how often the alter complimented or criticized the ego for exercise. Instrumental support was assessed by how often the alter took over chores for the ego so she could exercise, bought the ego equipment or gear for exercise, cared for the ego’s children or elderly parents so she could exercise, provided the ego transportation so she could exercise, and exercised with the ego. Response options were on a 5-point Likert scale ranging from never to always. For analysis, each type of support was examined.

#### Physical activity

The 7-Day Physical Activity Recall Interview (PAR) and ActiGraph GT3X accelerometers were used to measure minutes per week of MVPA of the ego at the baseline and 12 month visits. The PAR is an interviewer-administered self-report measure that asks about physical activity over the past week in at least ten-minute bouts [43]. Accelerometers were worn for 7 days and data was processed using the ActiLife 5 software, with a count cut point of 1952 to establish the minimum threshold for moderate intensity activity and minimum activity duration of 10 minutes. The 7-Day PAR and accelerometer measure different aspects of physical activity. Unlike the 7-Day PAR, accelerometers do not accurately estimate activities such as stationary bicycling, elliptical training, swimming, and upper extremity movement.

### Analysis

Analyses were conducted on 102 participants of both study groups with physical activity data collected at baseline and 12 months as well as with egocentric network data collected at 12 months. Descriptive statistics summarized participant baseline characteristics. Social network variables (structure, composition, tie strength, homogeneity, and support for physical activity) were calculated using UCINET and E-Net software (Analytic Technologies: Lexington, KY).

Ego-alter similarities on demographics and physical activity behaviors were measured by the E-I index [44]. The E-I index is calculated by the equation (*E-I)/(E+I)*, where *E* (external) is the number of alters different from the ego and *I* (internal) is the number of alters same as the ego. An E-I index score of -1 indicates the ego is tied only to similar others (complete homogeneity) whereas an E-I index score of +1 indicates the ego is tied only to different others (complete heterogeneity).

Multivariate linear regression models tested the relationship between social network variables and minutes per week of participant MVPA. Models testing the relationship between social network structure (all alters) and participant MVPA included network size or network size and density, transitivity or components as predictors. Models testing the relationship between social network composition and participant MVPA included network average age, education, and employment as well as proportion of females, Latinos, and familial ties as predictors. Models testing the relationship between social network tie strength and participant MVPA included network average relationship duration, living proximity, and emotional closeness as well as proportion of network with daily in-person contact as predictors. Models testing the relationship between social network physical activities and participant MVPA included proportion of network that engaged in walking, dancing, aerobic exercise, running, bicycling, swimming, and sports as predictors. Models testing the relationship between ego-alter similarities (homogeneity) and participant MVPA included E-I indices for composition characteristics or physical activity behaviors as predictors. Models testing the relationship between network support and participant MVPA included network average informational support, emotional support, or instrumental support as predictors. Change was modeled as residualized change where 12 month MVPA is conditional on baseline MVPA. Models controlled for study group and participant baseline MVPA and characteristics (age, income, education, employment status, and marital status). Statistical analyses were performed using PASW Statistics 21 (SPSS, Chicago, IL).

## Results

### Participant characteristics

The sample’s baseline characteristics are presented in Table 1. Participants had an average age of 40 ± 10 years. Most had at least a high school education (84%) and employment (53%). Most participants lived in households earning less than $40,000 annually (87%). The majority of participants were born outside the United States (82%) and Spanish-language dominant (57%). Most participants were married (64%) and had children under the age of 18 years living at home (72%). On average, participants engaged in less than ten minutes per week of MVPA based on self-report and more than thirty minutes per week of MVPA based on objective measurement.

**Table 1:** Participant (N = 102) baseline characteristics.

	N (%)
Age (years)	
18–35	29 (28)
36–50	56 (55)
>51	17 (17)
Education	
Less than high school graduate	16 (16)
High school graduate	47 (46)
College graduate	24 (23)
Graduate or professional school graduate	15 (15)
Employment	
Employed at least part-time	54 (53)
Income	
<$20,000	44 (43)
$20,000–39,999	45 (44)
≥$40,000	13 (13)
Nativity	
Foreign-born	83 (82)
Years of U.S. residence (mean ± s.d.)	15.2 ± 10.6
Language read and speak	
Only Spanish or more Spanish than English	58 (57)
Marital Status	
Married or living with partner	65 (64)
Children at home	
Under 18 years old	73 (72)
MVPA (minutes/week; mean ± s.d.)	
7-Day PAR	9.3 ± 16.0
Accelerometer	33.1 ± 71.8

### Network structure

Network structure characteristics based on all alters are presented in Table 2. The mean network size was 4 ± 2 (Table 2). The number of alters named ranged from 1 to 13. Networks were high in density (0.78) and transitivity (0.75) and low in components (1.2 ± 0.5).

**Table 2:** Network characteristics.

	All alters	Primary alters*
**STRUCTURE**		
Size (mean ± s.d.)	4.2 ± 2.1	
Density (mean)	0.78	
Transitivity (mean)	0.75	
Components (mean ± s.d.)	1.2 ± 0.5	
**COMPOSITION**		N (%)
Age (years)		
<18		12 (4)
18–35		90 (31)
36–50		104 (36)
≥51		84 (29)
Gender		
Female		180 (62)
Ethnicity		
Latino		258 (89)
White		28 (10)
Other		4 (1)
Language of communication		
Spanish		243 (84)
Relationship type		
Family		202 (70)
Child		48 (24)
Spouse		46 (23)
Sibling		42 (21)
Parent		29 (14)
Other		37 (18)
Friend		85 (29)
Coworker or neighbor		3 (1)
Education		
Less than high school graduate		51 (18)
High school graduate		137 (47)
College graduate		76 (26)
Graduate or professional school graduate		26 (9)
Employment		
Not employed		13 (5)
Student		27 (9)
Retired		16 (6)
Housewife		41 (14)
Service		85 (29)
Clerical		50 (17)
Professional		58 (20)
**TIE STRENGTH**		
Relationship duration		
Years known (mean ± s.d.)		21.8 ± 10.1
Living proximity		
Same household		91 (31)
Same neighborhood		25 (9)
Same city		66 (23)
Different city		40 (14)
Different county		11 (4)
Different state		12 (4)
Different country		45 (15)
In-person contact		
Daily		115 (39)
Weekly		81 (28)
Monthly		29 (10)
Less than monthly		63 (22)
Never		2 (1)
Emotional closeness		
Extremely		154 (53)
Very		94 (32)
Moderately		37 (13)
Somewhat		5 (2)
Not at all		0

*Closest three alters reported

### Network composition

Alter attributes are presented in Table 2. Alters were on average 41 ± 10 years old. Networks were primarily composed of females (62%), Latinos (89%), Spanish-speakers (84%), and family members (70%). Family members were mostly children, spouse, and siblings. The majority of alters had at least a high school education (82%) and were employed (66%).

### Ego-alter tie strength

Ego-alter ties were strong (Table 2). Egos reported knowing alters an average of 21 ± 10 years. Most alters lived in close proximity to the ego; in the same household (31%), neighborhood (9%), or city (23%). Few alters lived out of state (4%) or country (15%). Egos had in-person contact with most alters daily (39%) or weekly (28%). Egos characterized their relationship with the majority of alters as either extremely (53%) or very close (32%).

### Network homogeneity

Almost half of alters resembled egos in age or education (Table 3). The majority of alters were female and Latino and the small E-I indices indicate high homogeneity in these characteristics. Ego and alters were least similar in employment type.

**Table 3:** Network homogeneity.

	Ego (%)	Ego-Alter Similarity (%)	E-I
**COMPOSITION**			
Age		47	0.05
Gender		60	-0.19
Ethnicity		84	-0.68
Education		42	0.15
Employment		15	0.68
**PHYSICAL ACTIVITIES**			
Walk	78	54	-0.08
Dance	52	57	-0.14
Aerobics	46	56	-0.12
Run	39	59	-0.19
Bike	31	62	-0.25
Swim	17	74	-0.48
Sports	5	85	-0.69

Egos predominately engaged in walking for physical activity (Table 3). About half reported dancing or aerobic activities. Running and bicycling were less common activities. Swimming and playing sports were least common. Ego and alters were concordant in types of physical activities performed. The small E-I indices indicate high ego-alter homogeneity in engagement in swimming and sports.

### Network support for physical activity

The majority of alters provided egos with informational support and emotional support for physical activity in the form of advice (63%) and compliments (77%), respectively. Egos reported receiving criticism for exercise from some alters (16%). Less than a third of alters provided instrumental support such as taking over chores, buying equipment or gear, caring for children or elderly parents, or providing transportation so the ego could exercise. Participating in exercise with the ego was the most common form of instrumental support provided by alters (42%).

### Network variables and MVPA

Network structure was associated with MVPA (Table 4). Network size predicted increased minutes per week of MVPA. The relationship was only found for self-reported MVPA. The relationship between network density, transitivity, or components and MVPA was not statistically significant.

**Table 4:** Network structure & participant MVPA minutes per week.

	Self-reported MVPA				Objective MVPA			
	*Model 1*	*Model 2*	*Model 3*	*Model 4*	*Model 1*	*Model 2*	*Model 3*	*Model 4*
Size	9.20* (4.50)				4.56 (3.99)			
Density		27.48 (38.09)				19.89 (33.55)		
Transitivity			27.12 (29.61)				36.12 (28.67)	
Components				-23.80 (18.61)				-10.13 (16.68)

N = 102 participants

Models adjusted for study group, baseline characteristics, and network size.

Unstandardized regression coefficients (SE)

*P≤0.05

Few network composition variables were associated with MVPA (Table 5). Higher E-I for age (i.e., lower ego-alter similarity in age) predicted increased minutes per week of self-reported MVPA. Although having a greater proportion of alters that were family members was not associated with MVPA, examining each family member type revealed that having more alters that were children was associated with self-reported (ß = 0.603, SE = 0.28, *p* = 0.037) and objectively (ß = 0.511, SE = 0.25, *p* = 0.044) measured MVPA.

**Table 5:** Network variables & participant MVPA minutes per week.

			HOMOGENEITY (E-I Index)	
	Self-reported MVPA	Objective MVPA	Self-reported MVPA	Objective MVPA
**COMPOSITION**				
Age	-29.13 (17.61)	-29.00 (15.99)	46.59* (14.61)	17.90 (13.76)
Gender	-0.01 (0.38)	-0.05 (0.34)	22.35 (17.94)	7.64 (16.83)
Ethnicity	-0.23 (0.59)	-0.45 (0.52)	-8.51 (19.93)	3.10 (18.57)
Education	27.26 (19.48)	6.17 (18.38)	16.08 (16.42)	9.64 (15.26)
Employment	-1.21 (9.85)	6.06 (8.79)	20.85 (22.27)	9.19 (20.25)
Relationship type	0.58 (0.37)	0.41 (0.33)		
**TIE STRENGTH**				
Relationship duration	-0.36 (1.19)	-1.32 (1.03)		
Living proximity	9.00 (10.13)	8.63 (8.69)		
Contact frequency	0.32 (0.46)	0.82* (0.39)		
Emotional closeness	23.03 (19.94)	-1.27 (16.77)		
**PHYSICAL ACTIVITIES**				
Walk	-0.07 (0.35)	-0.48 (0.29)	-30.94* (15.17)	-13.36 (13.93)
Dance	0.00 (0.37)	0.00 (0.31)	-7.79 (14.81)	-8.73 (13.52)
Aerobics	-0.14 (0.40)	0.07 (0.35)	14.83 (14.03)	-2.86 (13.02)
Run	0.78* (0.37)	0.23 (0.32)	46.62* (13.98)	24.09 (12.94)
Bike	0. 24 (0.41)	1.04* (0.36)	15.60 (13.53)	19.69 (12.57)
Swim	0.88* (0.40)	0.10 (0.35)	-1.02 (14.55)	-11.82 (13.22)
Sports	-0.61 (0.48)	-0.48 (0.42)	-2.56 (17.95)	-23.08 (16.48)

Models adjusted for study group and baseline characteristics.

Unstandardized regression coefficients (SE)

*P≤0.05

Tie strength was associated with MVPA (Table 5). Contact frequency, specifically, daily in-person contact between ego and alters, predicted objectively measured MVPA. Relationship duration, living proximity, and emotional closeness were not statistically significant predictors of MVPA.

Ego-perceived alter physical activity behaviors were associated with MVPA (Table 5). Having a greater proportion of alters who engaged in running or swimming predicted increased minutes per week of self-reported MVPA. Having more alters who engaged in bicycling predicted increased minutes per week of objectively measured MVPA. Lower E-I for walking (i.e., higher ego-alter similarity in walking) and higher E-I for running (i.e., lower ego-alter similarity in running) were significantly associated with increased self-reported MVPA.

Network support for physical activity was associated with MVPA (Table 6). Emotional support and instrumental support predicted increased minutes per week of MVPA. Specifically, having alters that gave compliments, took over chores, or co-participated in exercise predicted more self-reported MVPA. Only the association with co-participation in exercise was statistically significant for both self-reported and objectively measured MVPA.

**Table 6:** Network support & participant MVPA minutes per week.

	Self-reported MVPA					Objective MVPA		
	*Model 1*	*Model 2*	*Model 3*	*Model 4*	*Model 1*	*Model 2*	*Model 3*	*Model 4*
**Informational support**								
Advice	18.17 (10.77)				13.81 (9.86)			
**Emotional support**								
Compliments		37.67* (8.60)				8.76 (8.36)		
Criticism		-26.92 (15.59)				-9.48 (14.77)		
**Instrumental support**								
Chores			55.98* (17.75)				28.41 (16.93)	
Equipment or gear			19.62 (17.72)				6.41 (16.78)	
Child or elderly care			-18.80 (14.87)				-10.96 (13.70)	
Transportation			-16.74 (14.12)				-3.27 (12.99)	
Exercise with you				43.11* (14.05)				32.09* (12.54)

Models adjusted for study group and baseline characteristics.

Unstandardized regression coefficients (SE)

*P≤0.05

## Discussion

This study examined the relationship between social network characteristics and physical activity in Latinas. Several social network characteristics predicted changes in physical activity. The results help advance the understanding of potential social influence on intervention physical activity outcomes.

Women with larger social networks achieved greater increases in MVPA than those with smaller networks. Other studies have shown that Latinos who are more socially connected walk more steps a day [45] and meet the physical activity recommendation for MVPA [12]. Having more social ties may provide a wider pool of people from which to enlist support. Because larger networks tend to be less dense, it may also indicate relaxed norms or receptiveness to physical activity. This finding suggests that being connected to more people may help promote adoption of physical activity among previously underactive Latinas in a lifestyle intervention.

Whereas cross-sectional studies have linked familial ties to lower physical activity levels and friendship ties to higher physical activity levels [20, 21, 25], this study did not find this relationship with MVPA change. In fact, women with more children in their network gained more MVPA than those with fewer children. Greater dissimilarity in age between women and their social ties also predicted more self-reported and objectively measured MVPA. Although the majority of women had under aged children living at home, network children were predominately adults. Hence, adult children may offer some advantage to engaging in physical activity.

Stronger social ties were associated with greater physical activity. Women who had daily in-person contact with more of their social ties increased more in objectively measured MVPA than those who had fewer social ties with daily in-person contact. In-person contact provided a unique benefit considering that other indicators of relationship strength were not related to MVPA change. Frequent in-person interactions may provide more opportunities for fostering support and norms for physical activity through sharing of information and behaviors among social ties. Providing in-person experiences may still serve as a useful strategy to promote physical activity among some groups especially when considering that while there has been growing interest in online social network-based physical activity interventions there are attrition and engagement challenges with the modality [46].

Women connected to physically active individuals engaged in more physical activity. This finding is consistent with studies on non-Latinas showing that physical activity is concordant among family members and friends [47–49]. Individuals with higher physical activity levels have been found to be embedded in networks consisting of others that regularly exercise [9, 10]. Data from longitudinal cohort studies reveal that physical activity behaviors are transmitted across social networks overtime suggesting behavioral influence [48, 50] Physical activity interventions for Latinos have attempted to leverage social network influence on physical activity but mainly in the form of social support through walking buddies or clubs and structured exercise classes [51–55]. For example, in *Fe en Accion* members of the community (promotoras) led exercise classes for churchgoing Latinas and significant increases were found in self-reported and objectively measured MVPA after 12 months [51]. Use of role models and group-focused programs may build networks that reinforce physically active lifestyles.

Our results showing that network support for physical activity was associated with greater changes in MVPA is in line with previous intervention studies [56, 57]. Alleviation of household tasks and co-participation in exercise were likely possible because social ties consisted predominately of family members who lived in close proximity. Having others to exercise with has been linked to physical activity in Latinos such that those who exercise with others engage in more leisure-time physical activity than those who exercise alone [22, 58]. The current study results, along with previous findings that showed exercising with others is more common in Latinos compared to non-Hispanic Whites [22], speak to the potential saliency of network-based physical activity programs for Latinas. Shaping these networks to enact and reciprocate emotional and instrumental support could potentially allow for longer term maintenance of physical activity.

This study has limitations. First, our sample consisted of predominately Mexican-American women participating in a lifestyle intervention for underactive women who completed the 12 month study assessment and thus may not represent other Latinas. Second, egocentric network data were used, which relies on the perception of respondents and may not be accurate. However, this approach is appropriate to understand to what extent networks may influence respondents. Additionally, assessments of the primary (closest) three alters tend to have higher reliabilities and validities [59]. Third, data collection was restricted for some information to the primary three alters to reduce participant burden. This allowed us to collect richer data although on a network subsample. Fourth, the sample size was modest and may have lacked adequate power to detect some effects. Finally, although physical activity data were longitudinal, the network data were cross-sectional and the directionality of relationships is not known. It is possible that changes in physical activity may lead to changes in network characteristics. For example, individuals who can modify their social network to better support their lifestyle change may be more likely to be physically active than those who cannot. Intervention studies should consider collecting longitudinal social network data at each outcome measurement. Assessing social networks overtime can also capture possible amplifying or “spill-over” intervention effects on individuals socially connected to participants enrolled in the intervention program.

There are also strengths to this study. This is one of the first studies to assess the relationship between social network characteristics and physical activity change in Latinas. To date, most research on social environment and physical activity has focused on adolescent networks or adult social support. Comprehensive data were collected to study various social network constructs. In addition, both self-reported and objectively measured physical activity were assessed which provides information on different aspects of physical activity. Specifically, measurement of self-reported physical activity allowed us to study social network predictors of behaviors such as swimming that are not measured via accelerometry. Moreover, measuring physical activity overtime allowed examination of changes in physical activity which has implications for lifestyle interventions.

## Conclusion

Social network structure, composition, tie strength, homogeneity, and support predicted physical activity change in Latinas. Network level predictors provide social context that can inform intervention strategies for the purpose of leveraging interpersonal influences on physical activity. To promote social norms and behavioral reinforcement of physical activity, future physical activity interventions targeting Latinas should consider programs that: 1) facilitate social interconnectivity and expansion of social networks, 2) include existing networks with emphasis on intergenerational family relationships, 3) provide opportunities for in-person interactions and joint activities with others, 4) allow exposure to peers and role models with diverse physical activity experience and skills, and 5) teach individuals and their networks how to elicit and provide effective emotional and instrumental support.

## Supporting information

S1 File Egocentric network questionnaire. (PDF)
