# Supplementary material for: Egocentric networks and physical activity outcomes in Latinas
Source: PLoS One. 2018 Jun 18;13(6):e0199139. doi: 10.1371/journal.pone.0199139 (PMC6005572; doi:10.1371/journal.pone.0199139)
Supplement: S1 File — (PDF) [file pone.0199139.s001.pdf]

Egocentric Network Questionnaire
(Interview)

Think of the people with whom you discussed important matters over the past 12 months.

This may include family or friends that may or may not live with you.

You may need to make your *best guess* in order to answer some of the following questions.

First, please list these people by first name starting with those you feel closest to and ending with those you feel least close.

Social network composition and tie strength

	Name 1	Name 2	Name 3
How old is [Name]?			
Is [Name] a man or woman?	Man Woman	Man Woman	Man Woman
What is the ethnicity of [Name]?	Latino<br>White<br>Other:_________	Latino<br>White<br>Other:__________	Latino<br>White<br>Other:__________
What language do you usually speak with [Name]?	English<br>Spanish<br>Other:__________	English<br>Spanish<br>Other:__________	English<br>Spanish<br>Other:__________
How do you know [Name]?	Family:_________<br>Friend<br>Other:_________	Family:_________<br>Friend<br>Other:__________	Family:_________<br>Friend<br>Other:__________
What is the highest level of education completed by [Name]?	Less than H.S. graduate<br>High school graduate<br>College graduate<br>Graduate or professional school graduate	Less than H.S. graduate<br>High school graduate<br>College graduate<br>Graduate or professional school graduate	Less than H.S. graduate<br>High school graduate<br>College graduate<br>Graduate or professional school graduate
What is [Name] type of employment?			
How many years have you known [Name]?			
Where does [Name] live?	Same household<br>Same neighborhood<br>Same city<br>Different city<br>Different county<br>Different state<br>Different country	Same household<br>Same neighborhood<br>Same city<br>Different city<br>Different county<br>Different state<br>Different country	Same household<br>Same neighborhood<br>Same city<br>Different city<br>Different county<br>Different state<br>Different country
How often do you see [Name] in-person?	Never<br>Less than Monthly<br>Monthly<br>Weekly<br>Daily	Never<br>Less than Monthly<br>Monthly<br>Weekly<br>Daily	Never<br>Less than Monthly<br>Monthly<br>Weekly<br>Daily
How close do you feel to [Name]?	Not at all close<br>Somewhat close<br>Moderately close<br>Very close<br>Extremely close	Not at all close<br>Somewhat close<br>Moderately close<br>Very close<br>Extremely close	Not at all close<br>Somewhat close<br>Moderately close<br>Very close<br>Extremely close

Social network structure

Does [Name] know [Name]?

	Name 1	Name 2	Name 3
**Name 1**			
**Name 2**	No Yes		
**Name 3**	No Yes	No Yes	

You may need to make your best guess to answer the following questions.

Ego and alter physical activity behaviors

How often did [Name] do the following activities?

	Never	Rarely	Some of the time	Most of the time	Always
Walk for leisure	☐	☐	☐	☐	☐
Run or jog	☐	☐	☐	☐	☐
Ride a bicycle or stationary bike	☐	☐	☐	☐	☐
Swim	☐	☐	☐	☐	☐
Dance	☐	☐	☐	☐	☐
Do other aerobic exercise (e.g., elliptical, zumba, exercise games)	☐	☐	☐	☐	☐
Play team sports (e.g., soccer, basketball, baseball)	☐	☐	☐	☐	☐

How often did you do the following activities?

	Never	Rarely	Some of the time	Most of the time	Always
Walk for leisure	☐	☐	☐	☐	☐
Run or jog	☐	☐	☐	☐	☐
Ride a bicycle or stationary bike	☐	☐	☐	☐	☐
Swim	☐	☐	☐	☐	☐
Dance	☐	☐	☐	☐	☐
Do other aerobic exercise (e.g., elliptical, zumba, exercise games)	☐	☐	☐	☐	☐
Play team sports (e.g., soccer, basketball, baseball)	☐	☐	☐	☐	☐

Social support and physical activity

How often did [Name] do the following?

	Never	Rarely	Some of the time	Most of the time	Always
Give you advice on exercising	☐	☐	☐	☐	☐
Compliment you for exercising	☐	☐	☐	☐	☐
Criticize you for exercising	☐	☐	☐	☐	☐
Take over chores so that you could exercise	☐	☐	☐	☐	☐
Buy equipment for you to exercise	☐	☐	☐	☐	☐
Take care of your children/elderly parents so you could exercise	☐	☐	☐	☐	☐
Provide transportation for you so you could exercise	☐	☐	☐	☐	☐
Exercise with you	☐	☐	☐	☐	☐
